# Economic Diversification Trends in the Gulf: the Case of Saudi Arabia

**DOI:** 10.1007/s43615-021-00106-0

**Published:** 2021-08-28

**Authors:** Sarah Muhanna Al Naimi

**Affiliations:** Gulf Studies Program, Qatar University, P O 2713 Doha, Qatar

**Keywords:** Rentier state, Knowledge-based economy, Economic diversification, UN 2030 Sustainable Development Goals (SDGs)

## Abstract

A national economy which is dependent on income from just one source is vulnerable, especially when that income comes from non-renewable resources. The sustainable prosperity of an economy thus relies on the successful implementation of economic diversification. Diversification is key to creating an attractive, flourishing environment in a country and improving the quality of its institutions and its citizens’ lives. The countries of the Gulf Cooperation Council (GCC) are accelerating their efforts to achieve economic diversification, with their national visions reflecting a shared aim of securing permanent high standards of living for future generations. After the first boom in oil prices in 1970, Saudi Arabia’s government introduced primary development plans to diversify its economy. In 2016, it announced its 2030 vision to establish sustainable growth through economic diversification. The economic diversification strategy of Saudi Arabia is founded on several pillars, including investment in human capital and education and investment in non-oil sectors such as tourism. This paper aims to analyze the economic diversification trends in the GCC region with a special focus on Saudi Arabia as a case study. Within this wider context, the paper will concentrate on Saudi Arabia’s efforts to achieve diversification by building a knowledge-based economy. Focusing on the quality of education and research improves the human capital available in the country which contributes to the growth of the economy. Results reveal that although Saudi Arabia has embarked on its diversification plans, the current status of oil prices, the deficit in the Saudi general budget, and the country’s traditional educational system will hinder and slow this process.

## Introduction

In the early years of the 1930s, the discovery of oil in the GCC region represented a turning point for the six sheikhdoms: Kuwait, Qatar, Bahrain, UAE, Oman, and Saudi Arabia. Once composed of hermetic, nomadic, and illiterate communities, they have transformed into states that are characterized by major growth and high energy consumption. As the control of the oil industry moved to the GCC sheikhdoms under the process of nationalization, these monarchies initially fixed the prices of energy products and services at a low level. The increase in oil prices led to an increase of oil revenues and the consolidation of autocratic regimes. Oil was considered by these ruling elites as the core of their regimes’ existence and the foundation of their economic prosperity. Since these countries were founded on a patronage system, heavily reliant on the use of subsidies, a new social contract was needed between the GCC rulers and their populations which was based on rents. That is, rulers would distribute parts of the rents emanating from oil revenues to their subjects/citizens in return for the consolidation of popular support for the ruling sheiks. Hvidt [1] labels the GCC countries “allocation states.” Some examples of these rents are favors, public goods, and services.

However, this process had its price. Despite the golden façade of these Gulf rentier states, the weakness of the system is apparent in the countries’ inability to withstand fluctuations in international oil prices. Hvidt [1] believes that the system is incapable of sustaining the necessary development of the GCC countries as the income it provides for their populations and economies is volatile. For almost half a century, the source of income for the GCC region has been firmly linked to hydrocarbon revenues; these non-renewable resources form the mainstay of their economies. Shifts in the prices of hydrocarbon resources therefore impact directly on these rentier states, undermining the strength of their patriarchal systems, as demonstrated when oil prices started to decline sharply in 2015 and 2016 [2]. Together with low domestic oil prices, subsidies led to the increase of local consumption of energy resources to the extent that domestic demand began to exert pressure on the GCC’s oil exports. These issues pose a threat to the GCC’s energy security. The importance and value of the Gulf region are clear: the GCC region possesses 30% of the world’s crude oil and 22% of the world’s gas [3]. These territories are witnessing dynamic shifts and influential changes in different realms—political, demographic, and economic. The GCC’s economic diversification represents a decisive solution to address this current risk.

This paper is organized as follows. It begins with a description of the methodology and the conceptual framework that underpin the study, including definitions of the key concept of economic diversification and other relevant terms. A sketch of the historical situation then provides an overview of the main economic diversification trends in the GCC states. The following two sections focus on the process of economic diversification in Saudi Arabia as outlined by its national Vision 2030 and analyze this economic diversification through the lens of efforts to establish a knowledge-based economy. The paper ends with some brief conclusions.

## Methodology

Examining and exploring diversification trends in the GCC, and in particular the trajectory of economic diversification in Saudi Arabia, is the principal goal of this paper. In addition, it studies the progress of this strategy and sheds light on the factors that have played a role in delaying its achievement. Economic diversification in Saudi Arabia started in 1970 and was further consolidated in April 2016 in the Saudi Vision 2030 [4]. In the words of Prince Mohammed bin Salman bin Abdulaziz, Saudi Arabia’s Crown Prince, this vision aims specifically “to reinforce and diversify the capabilities of our economy, turning our key strengths into enabling tools for a fully diversified future”.^1^

Research methodology is defined by Bhujanga Rao [5] as “an appropriate method or methods for an exhaustive and detailed investigative study of [the] problem of research” (p.10). This study falls within the category of analytical research, in which the researcher utilizes available data in order to make a critical evaluation of the selected research problem [5]. This study depends on documentary analysis. In understanding the development of diversification policy in Saudi Arabia, the analysis of documents gives insight into the components and actors in the progress of this policy as well as the linkages between policy and practice. Prior [6] considers document analysis to be essential in social sciences, where documents have a dual role: first, they contain written information which can be examined to interpret a social phenomenon; second, they can catalyze social interaction. Hence, studying documents can shed light on the context in which a certain policy emerges and how that policy influences and reflects societal change. Sources that inform this paper include documents of the Saudi government, the United Nations, the World Bank, and the World Economic Forum.

## Conceptual Framework

Achieving economic diversification has a positive impact on the sustainable growth and prosperity of a nation. To construct a comprehensive understanding of economic diversification, this paper will begin by defining the concept of economic diversification and scrutinizing the definitions of several related terms. In the context of the Gulf region, economic diversification refers to reducing the dependence of its economy on hydrocarbon resources such as oil and gas. Being dependent on a single source for revenue directly impacts the economy, linking it to the price of this principal source. In this region, consumption levels and population growth are putting increasing pressure on the depleted resources in the Gulf. The GCC countries are facing a future in which local consumption of energy resources will surpass the foreign demand for these resources, jeopardizing the whole basis of the region’s economy.

Economic diversification refers to the transformation of an economy that is reliant on a specific source for its income (oil and gas revenues as in the case of the GCC countries), into an economy in which other sectors, including non-hydrocarbons, play a significant role. Examples of non-hydrocarbon fields might include industry, agriculture, and tourism. For the GCC states, economic diversification aims to minimize the risks which are associated with oil price fluctuations, by reducing their reliance on a single finite resource and instead dispersing it across multiple potentially infinite resources [7]. The expectation is that economic diversification will not only make the economy less vulnerable to risks associated with price swings in the short term, but it will also enable the economy to provide prosperity for the forthcoming generations when oil and gas reserves run out. In 2008, the state of Qatar published its Qatar National Vision 2030 which warns that a failure to implement this goal would amount to an injustice against future generations: “[t]he rights of future generations would be threatened if the depletion of non-renewable resources were not compensated by the creation of new sources of renewable wealth” [8]. In the same vein, Al-Jundi [7] discusses the necessity of transforming the economy of the UAE into one that “can survive without oil revenues stimulation and … maintain economic achievements after the end of the oil era” (p.15).

Economic diversification also paves the way for the accomplishment of the UN’s Sustainable Development Goals (SDGs). The SDGs comprise 17 goals which were agreed upon in 2015 by all UN member states, to be achieved by 2030, within the “2030 Agenda for Sustainable Development” [9]. The UN defines the SDGs as part of an action plan that aims to “end poverty, protect the planet, and ensure that by 2030 all people enjoy peace and prosperity” [9].

Economic diversification consolidates the economic and financial foundations of a country [7]. If one form of investment yields low revenues, other investment tools can compensate. Thus, diversification requires changes to be implemented in the economic infrastructure. These shifts are illustrated by an increase in manufacturing output, the expansion of the role of the private sector, and the creation of a circular economy and a knowledge-based economy. The expansion of manufacturing in terms of product diversity and output through successful industrialization results in the emergence of another level of diversification which is labeled by Morakabati et al. (2014, cited in [2]) as export diversification. Export diversification has been increasing recently in developing countries. In the course of the past half century, nearly 80% of countries that were importers of principal goods have transformed into exporters of manufactured goods. Export diversification boosts the economy by, for example, promoting the effective deployment of natural and human resources, attracting foreign investments, and furthering social and economic progress. Wilhelms [10] explains in detail the gains to be achieved from export diversification by developing countries. He argues that export diversification leads to economic progress as it stabilizes trade profits, creates new job opportunities, and broadens export processes. Clearly, diversification of exports would be significant for a region like the GCC, which imports nearly 98% of its needs from global markets [2]. By increasing exports, the GCC countries will encourage private sector engagement and will reduce the risk of reliance on oil and gas by shifting attention towards exporting manufactured and industrialized exports instead of only crude oil and natural gas.

The increased engagement of the private sector can be expected to lead to further economic diversification, for example, through a process of privatization and through Public–Private Partnership programs (PPPs) [11] which would pump money into the economy from sources outside the energy sector. The private sector would also bring foreign money into domestic markets through foreign direct investments—investments which would benefit the oil-based economies of the GCC countries and ease their transfer to knowledge-based economies. Hvidt [1] stresses that these investments can bring new employment opportunities, new technologies, and innovative methods of management to an economy. Continued dependence on the oil and gas sector cannot provide careers for the growing population in the long term, especially in a youthful region such as the GCC. According to Abyad [12], youth below the age of 25 years constitutes 54% of the population of the GCC. This issue can be addressed by engaging the private sector in creating a diversified economy and generating new job opportunities.

In this context, it should be noted that the public sector in the GCC region is currently the main source of employment. Levels of bureaucracy in this sector are high, with resultant high costs [13]. This can threaten the sustainability of GCC economic systems, which are dependent on non-renewable hydrocarbon resources. Kinninmont [14] argues that the revenues emanating from these economic systems cannot offset the increases in the public expenditure of these rentier states that have taken place since 2003. The World Bank’s “Global Economic Prospects” 2020 report supports this claim and adds that even GCC governmental investments did not help in counterbalancing the shrinking oil market activity worldwide [15]. Although some steps have been taken towards economic diversification and the establishment of sovereign wealth funds (SWFs) in GCC countries, these measures are still in the early stages, and the income generated by these SWFs is not yet enough to compensate for low oil prices on the international market. Sovereign wealth funds are investment funds owned by a state. Funding for SWFs emanates from different sources, including commodity exports [16] such as (in the case of the GCC region) hydrocarbon resources. The main reason for establishing SWFs in GCC countries has been to diversify their economies and secure sustainability for future generations, but Kinninmont [14] cautions that SWFs cannot provide financial stability that completely offsets GCC government spending nor can they counteract the challenges that result from depending on an unrenewable hydrocarbon resources. For example, Abu Dhabi’s SWF, which is considered one of the top global SWFs, would provide a partial substitution for only 9 years of this emirate’s public spending [14].

Investment in human capital, meanwhile, requires reform in the educational process to create specialized and highly qualified citizens. Mishrif and Al Balushi [2] point out that attempts to modernize education in several GCC countries such as Qatar and the UAE have focused only on investing in the latest physical infrastructures and not on the necessary curricular reforms. Traditional educational systems in the GCC region will thwart the process of creating specialized human capital. To achieve their goal, GCC countries must move from resource-based hydrocarbon economies into diversified and knowledge-based economies. The Organisation for Economic Co-operation and Development (OECD) defines the latter as “economies which are openly based on production, distribution, and use knowledge and information with the important role of information, technology and learning in performing” [17] (p. 168), as opposed to resource-based economies which are dependent on natural resources as the source of GDP income. The GCC economies currently fall within the latter type, but their diversification plans focus on circularity and establishing a knowledge-based economy.

Ozili [18] defines a circular economy as “an economic system that promotes efficiency through eliminating waste and the continuous use of resources” (p.3). A circular economy can enhance sustainability by shifting patterns of consumption and production through innovative strategies and creative business models. Unlike traditional linear economic systems which are based on extraction of raw materials, production, and disposal of waste, a circular economy aims at investing efficiently in the life cycle of resources. Thus, the loop in the production cycle is closed, recycling is maximized, and waste is minimized [18]. Shifting to a circular economy pattern promotes environmental sustainability through efficient waste management, energy recovery, and careful usage of raw materials. There is a correlation between a circular economy and a knowledge-based economy: in order to effect the transition to a circular economy, a knowledge-based economy which improves educational levels is essential. Al-Saidi et al. [19] describe a shift in knowledge and reform of institutions as key factors in creating economic incentives which support, and are supported by, a circular and knowledge-based economy. In establishing a knowledge-based economy, educational systems play a vital role: “investment in education will be the essential factor to enable the local manufacturers to be able to meet global standards” [13] (p.12) and to avoid being left behind in terms of global competitiveness. In the context of the GCC region, only two GCC states have managed to reach the top thirty countries in the World Competitiveness Report 2019: UAE, ranked 25th globally, and Qatar, ranked 29th globally [20]. Wirba [17] argues that the Gulf region has succeeded in creating GDP levels that are similar to those of the developed countries, but the levels of their investment in research and development (R&D) are similar to those of developing countries.

Economic diversification occurs in two directions—vertical and horizontal. Vertical diversification concentrates the process of expansion within a sector by introducing additional steps. Hvidt [1] argues that this form of diversification occurs in a backwards and forwards direction in the stages of an industry. This maintains earnings and increases value-added products domestically. In vertical diversification, an industry can shift itself from one stage into another [1]. In other words, there is a gradual change in the stages of one industry from primary to secondary to tertiary sector. At the time of writing, it can be observed that the industries of the GCC countries are still in the primary phase, which is the process of extraction of a raw material (e.g., a natural energy resource), and preparation for it to be exported. For GCC countries to upgrade their industries to reach the secondary and tertiary levels, these states must accelerate and deepen their investment in their local manufacturing industries. These industries would then be in a position to transform these extracted resources into tangible products, which represents the secondary sector. The tertiary sector would be represented by the processes of selling these manufactured outputs and transporting them. Furthermore, the GCC countries must also pay more attention to sectors that have so far been less of a focus, such as agriculture.

Horizontal diversification, on the other hand, entails the expansion of different possibilities for innovative products. Recently, the GCC countries have turned to this category of diversification as illustrated by their investments in non-hydrocarbon fields such as culture, tourism, and sport. Although there is a tendency to use the terms economic diversification and industrialization interchangeably, there is a difference. According to Hvidt [1], industrialization aims to increase income and profits, while the aim of diversification is to lower risk by spreading it over multiple sectors. Hvidt [1] suggests that the overlap in the use of the terms comes about because industrialization penetrates vertical diversification: there is an interdependent relation between the two processes. In vertical diversification, an increase of volume in manufacturing or service sectors occurs as a result of industrialization. Hvidt [1] defines industrialization as “the process of converting to a socioeconomic order in which industry is dominant” (p.6).

## A Historical Overview of Diversification in the Gulf Region

The Gulf region’s pre-oil economy depended mainly on the fishing industry and pearl diving trade. This region did not establish agriculture due to the harsh climate and the scarcity of the necessary natural resources. Krane [21] depicts the populations of the Gulf at that time as being small and struggling with poverty because the economy of the Gulf was undeveloped. However, Gulf populations managed to survive in this environment quite impressively. The discoveries of oil in the Gulf region from the 1930s onwards were a turning point for the fate of these territories. The region has been transformed into a modernized and urban hub. The trend towards economic diversification in the GCC region has increased as global oil prices have witnessed a downward trend since 2014. Between 2014 and 2018, oil prices fell by more than 50% from $110 to $50 per barrel [22]. According to Mishrif and Al Balushi [2], the price of a barrel of oil dropped as low as $27 in 2016.

Economic diversification started in the GCC region in the 1970s, during a period of high oil prices. At that time, Saudi Arabia embarked on its diversification plans to earn revenues from non-oil resources, focusing on fiscal revenues. Hvidt [1] criticizes Saudi Arabia because, in spite of having “the longest and most elaborate tradition of planning among the GCC countries, institutionalized in the Ministry of Economy and Planning (MoEP)” (p.28), it has not achieved any tangible outcomes. The UAE and Kuwait followed in Saudi Arabia’s footsteps in this period [23]. There is a long gap between these early diversification efforts by GCC countries and the latest attempts at diversification in the region. Luciani (2012, cited in [2]) argues that the GCC countries are slow in the implementation of their diversification strategies as they are dependent on inconsistent oil revenues: “hence there is no rush to disrupt their economic system as long as oil rents will continue to be the main source of government revenues for the foreseeable future” (p.2). It can be inferred that early steps of diversification in the 1970s in some GCC countries, as mentioned above, took a vertical direction, focusing on the hydrocarbon energy industry. Mishrif and Al Balushi [2] support this inference, indicating that huge investments at that time took place in the energy field and related industries such as petrochemicals, chemicals, fertilizers, and aluminum.

## Current Overview of the Saudi Economy

Since oil was first discovered in the region, the economy of Saudi Arabia has been dependent on oil revenues. Saudi Arabia possesses about 30% of global crude oil and is ranked second in terms of the global production of crude oil. It also has 22% of proven gas reserves, ranked sixth in the world [3]. Saudi Arabia’s general budget depends hugely on oil revenues. Euchi, Omri, and Al-Tit [22] expect that these resources will be depleted in the coming two decades. Non-oil revenues accounted for only 13% of the Saudi general budget in 2018. Saudi Arabia’s deficit budget is 4.6% of its total 2018 GDP [23], with the possibility that it could reach 6.4% or worse in the 2020 general budget. Certain changes have been adopted to curb unnecessary expenditure in order to adapt to risks related to the fall in global oil prices and the COVID-19 pandemic. These changes will have a direct effect on Saudi Arabia’s debts and will impact negatively on its foreign exchange reserves. Cahill [24] points out that Saudi Arabia issued $19 billion in debts in 2020 and ponders on the sustainability of the country’s foreign exchange reserve in light of its sharp decline by $27 billion.

This recent snapshot of the Saudi economy contrasts with the ambitious Saudi National Vision 2030 [4]. The essence of this vision is to reduce the reliance of the Saudi economy on oil revenues through economic diversification. The Saudi vision identified several pillars which are required for implementing a diversified and sustainable economy. These pillars are founded on the creation of a knowledge-based economy, encouraging entrepreneurship and enhancing private sector participation, achieving gender equality in the domestic job market, and investment in non-oil sectors such as tourism [4]. The following section will focus on the country’s progress in one of these aspects: the creation of a knowledge-based economy.

## Creating a Saudi Knowledge-Based Economy

If creating a knowledge-based economy is the cognitive bridge to creating a diversified economy, then educational reform and investment in R&D provide the infrastructure for building that knowledge-based economy, which would enable Saudi Arabia to transition away from an oil-based economy. Wirba [17] argues that success in building a knowledge-based economy represents the basis for improved global competitiveness. Saudi Arabia was ranked 34th in the 2019 Global Competitiveness Report [20], whereas a country that is one of the G-20 economies should score a rank among the top 20 globally. One issue, according to Wirba [17], is that the GCC countries invest relatively little in R&D, despite their abundant economic and financial resources; Mishrif and Al Balushi [2] corroborate this finding, demonstrating that the GCC region invests less than 1% of its GDP in R&D.

As discussed above, constructing a knowledge-based economy requires that citizens are equipped with analytical skills and other key abilities that are demanded by the private sector and are needed to function in a globalized market. Improving the quality of education requires investment in education and the setting up of research institutions. This in turn demands a clear and measurable educational vision. If successful, educational reform will expand knowledge through unlimited access to information sources. Furthermore, it will distribute educational roles among multiple players, including the engagement of the private sector, working with the education ministry.

Saudi Arabia is the largest GCC country in terms of geography and population, which could be an asset in terms of building a knowledge-based economy; moreover, it is one of only two GCC countries in which nationals outnumber foreigners, accounting for 69% of the population [2]. To move towards implementing a knowledge-based economy, Saudi Arabia has launched reform programs to modernize its educational process, focusing especially on the higher education curriculum, to encourage a mentality of entrepreneurship to meet the demands of the job market (particularly in the private sector). Although these are promising steps towards building a knowledge-based economy, this is likely to be a long and thorny path. Saudi Arabia has a population of nearly 27 million people, of which Saudi citizens constitute 18.6 million. Although in the long run this might be an asset, the large population, together with the underdeveloped Saudi economy, has resulted in falling GDP in Saudi Arabia, even though it is one of the largest exporters of oil [1]. The majority of the population are currently equipped with a traditional education, which has led to problems such as high youth unemployment, a concentration of jobs offered in the public sector, and an increase in rates of poverty. The youth unemployment rate in Saudi Arabia is escalating, especially among females. In 2016–2017, Saudi youth unemployment reached 25% overall, with female youth unemployment reaching 46.3%. These figures challenge the goals which were set out by the Saudi National Vision 2030 [4], which include reducing the rate of unemployment from 11.6 to 7% and increasing women’s engagement in the job market from 22 to 30%.

Cowan [25] estimates that between 2 and 4 million Saudis are living in poverty with a daily income of $17 or less. Mishrif and Al Balushi [2] highlight several factors that thwart the remodeling of Saudi education and help perpetuate the gap between private-sector job requirements and the qualifications of potential candidates. They argue that the low-quality education offered by Saudi educational institutions plays a role in the huge numbers of unemployed youth; they also draw attention to the absence of a clear educational vision for delivering innovation and encouraging improved analytical skills among Saudi students. Furthermore, the burdensome bureaucracy, corruption, and lack of innovation of Saudi educational institutions add to these problems. Altorki [26] describes how the state in Saudi Arabia interferes with the curriculum and data with the help of the religious authority “Ulama,” which limits availability of and access to information. Altorki [26] describes the resulting pedagogy as “repetitive, redundant, synoptic, synthetic and methodologically narrow” (p.242).

## Conclusion

The principal aim of economic diversification is to make the economy resilient and robust. A strategy of economic diversification can sustain economic growth and minimize the risks of over-reliance on one sector. Recently, the GCC countries have started to look beyond their dependence on oil revenues, with ambitious national visions reflecting their plans for economic diversification. Saudi Arabia’s National Vision 2030 [4] declared that the Saudi economy would be reformed through economic diversification. To achieve this, several pillars were identified as the basis for transforming the Saudi economy in order to reduce the risks associated with reliance on oil revenues. One of these pillars is the establishment of a knowledge-based economy. The assessment undertaken in this article suggests that the path will not be smooth. Oil remains the main engine of the Saudi economy. Given the volatility of global oil prices, the growing deficit in the Saudi general budget, and domestic challenges such as increasing numbers of unemployed youth and an unreformed educational system, the process of economic diversification in Saudi Arabia is likely to suffer from setbacks along the way.

## Footnotes

1 See https://www.vision2030.gov.sa/v2030/leadership-message/
